# Augmented reality- virtual reality wartime training of reserve prehospital teams: a pilot study

**DOI:** 10.1186/s13584-024-00634-8

**Published:** 2024-09-12

**Authors:** Arielle Kaim, Efrat Milman, Eyal Zehavi, Amnon Harel, Inbal Mazor, Eli Jaffe, Bruria Adini

**Affiliations:** 1 Department of Emergency and Disaster Management, Faculty of Medicine, School of Public Health, Tel Aviv University, Tel Aviv, Israel; 2 Surgical Science, Airport City, Israel; 3 Public Relations, Training and Volunteers Division, Magen David Adom, Tel Aviv, Israel

**Keywords:** Prehospital teams, Augmented reality, Virtual reality, Training, Intubation

## Abstract

**Background:**

In the realm of trauma response preparation for prehospital teams, the combination of Augmented Reality (AR) and Virtual Reality (VR) with manikin technologies is growing in importance for creating training scenarios that closely mirror potential real-life situations. The pilot study focused on training of airway management and intubation for trauma incidents, based on a Trauma AR-VR simulator involving reserve paramedics of the National EMS service (Magen David Adom) who had not practiced for up to six years, activated during the Israel-Gaza conflict (October 2023). The trauma simulator merges the physical and virtual realms by utilizing a real manikin and instruments outfitted with sensors. This integration enables a precise one-to-one correspondence between the physical and virtual environments. Considering the importance of enhancing the preparedness of the reserve paramedics to support the prehospital system in Israel, the study aims to ascertain the impact of AR-VR Trauma simulator training on the modification of key perceptual attitudes such as self-efficacy, resilience, knowledge, and competency among reserve paramedics in Israel.

**Methods:**

A quantitative questionnaire was utilized to gauge the influence of AR-VR training on specific psychological and skill-based metrics, including self-efficacy, resilience, medical knowledge, professional competency, confidence in performing intubations, and the perceived quality of the training experience in this pilot study. The methodology entailed administering a pre-training questionnaire, delivering a targeted 30-minute AR-VR training session on airway management techniques, and collecting post-training data through a parallel questionnaire to measure the training’s impact. Fifteen reserve paramedics were trained, with a response rate of 80% (*n* = 12) in both measurements.

**Results:**

Post-training evaluations indicated a significant uptick in all measured areas, with resilience (3.717±0.611 to 4.008±0.665) and intubation confidence (3.541±0.891 to 3.833±0.608) showing particularly robust gains. The high rating (4.438±0.419 on a scale of 5) of the training quality suggests positive response to the AR-VR integration for the enhancement of medical training,

**Conclusions:**

The application of AR-VR in the training of reserve paramedics demonstrates potential as a key tool for their swift mobilization and efficiency in crisis response. This is particularly valuable for training when quick deployment of personnel is necessary, training resources are diminished, and ‘all hands on deck’ is necessary.

## Introduction

In a time when the risk of disasters, whether natural or human-induced, is a constant concern, the importance of Emergency Medical Services (EMS) in delivering timely and effective care to affected individuals is crucial [1, 2]. The sudden onset of these threats imposes a significant strain on prehospital systems, necessitating rapid adaptation and the development of a capable and responsive emergency medical team. The activation of reinforcing responders becomes essential to augment the system’s capacity in such times of need. With active personnel critically occupied on the front lines, it is imperative for EMS infrastructures to adopt innovative and high-caliber training methods to expedite the upskilling of their workforce. These initiatives are crucial to swiftly amplify the operational readiness of the reinforcement teams, preparing them to adeptly handle various emergency scenarios and fortify an emergency response system ready to be called into action.

Magen David Adom (MDA), as Israel’s primary prehospital emergency medical service, is often the first to respond to a spectrum of health and security emergencies across the nation [1, 2]. Nevertheless, despite the extensive experience in handling Mass Casualty Incidents (MCIs) and rigorous preparedness drills, the Emergency Medical Services faced an unparalleled emergency on October 7, 2023. Hamas, from the Gaza Strip, initiated a coordinated assault on southern and central Israel. This offensive began with bombardment of rockets aimed at civilian areas and included intrusions near the Gaza border. The incident escalated into broad clashes involving civilians, security personnel, medical teams, and the Israel Defense Forces. The repercussions of this mega-MCI were significant, with ongoing developments. Recent reports indicate that there were over 1,400 Israeli deaths and around 5,600 injuries, classifying it as one of the most lethal terrorist attacks in modern times [3]. Moreover, reports confirm that at least 240 people, including 30 children, were abducted to Gaza, intensifying the gravity of the aftermath. On that day, MDA experienced intentional assaults on their ambulances and staff, resulting in multiple fatalities and injuries within their ranks. MDA personnel responded to hundreds of emergency calls, delivering medical aid during the events that began on October 7th and saved numerous lives [3]. These events precipitated the onset of a full-scale war.

To address crises effectively, MDA has implemented a strategy similar to military protocols. This involves mobilizing reserve paramedic volunteers to strengthen its prehospital system based on identified needs during times of crisis. In October 2023, MDA activated additional resources in areas anticipating paramedic shortages. This action was taken due to paramedics being called to reserve military service and based on risk assessments indicating where needs were most likely to exist, with higher recruitment focused on the South of Israel at that time of this study. This measure is a proactive step to enhance the emergency response network and address arising threats efficiently. In the midst of the crisis, a significant number of the reserve volunteers had not engaged in medical practice for an extended period of time (between one to six years of no training and volunteering) and, therefore, necessitated refresher training before they could be reintegrated into the prehospital emergency system.

Virtual Reality (VR) and Augmented Reality (AR) technologies have emerged as valuable tools for enhancing professional competencies in medical training, allowing active duty personnel to remain in service instead of attending traditional training sessions. VR immerses users in a fully digital environment, creating a simulated experience that attempts to block out the real world. In contrast, AR overlays digital information and objects onto the real world, enhancing the user’s perception of their environment. Although these technological tools have long been associated with the gaming industry and other fields, they have not yet been fully utilized for advanced technical training within the healthcare sector, as noted by Lochmannova et al. (2022) [4]. In the realm of trauma response preparation for prehospital teams, the combination of Augmented Reality (AR) and Virtual Reality (VR) with manikin technologies is growing in importance for creating training scenarios that closely mirror potential real-life situations, also known as Mixed Reality [5]. These advanced AR-VR simulation programs are designed to provide a comprehensive sensory learning space, heightening the realism of scenarios in which emergency interventions and critical decision-making are practiced. Augmented Reality (AR) and Virtual Reality (VR) bridge between the digital and physical realms. Medical professionals gain from the opportunity to engage in intricate procedures with real-time feedback, advancing their skills in an environment free of risk.

In the critical context of emergency medical care, a paramedic’s expertise extends beyond mere technical know-how, with perceptual elements like self-efficacy and resilience playing a pivotal role. These psychological facets can significantly shape the speed and precision of decision-making, as well as the overall effectiveness of patient treatment [6, 7]. The objective of this research was to evaluate how training with a Trauma AR-VR simulator influences these key perceptual factors among reserve paramedics.

## Methods

### Study design

Considering the importance of enhancing the preparedness of the reserve paramedics to support the prehospital system in Israel, an intervention study was conducted in October 2023, following the onset of the Israel-Gaza war. The study focused on training airway management and intubation for trauma incidents using a customized Trauma AR-VR simulator prototype developed by a leading provider of medical simulation technologies. The trauma simulator merges the physical and virtual realms by utilizing a real manikin and instruments outfitted with sensors. This integration ensures a precise one-to-one correspondence between the physical and virtual environments, allowing for real-time feedback during procedures, including vital signs affected by patient management and drug administration. The AR-VR simulator allows for the introduction of dynamic complications that may occur during intubation as well as a wide range of pre-hospital and hospital scenarios. This enables participants to adapt to different levels of intubation complexity and various environmental challenges. Moreover, the technology supports both proctored and self-directed training sessions, offering flexibility in training approaches. This study serves as a preliminary pilot study for the validation of the simulator. The study focused on training paramedic reserves in airway management (intubation) using a 30-minute module. This module simulated a war-related scenario from a previous conflict, where a wounded soldier required stabilization, including the performance of intubation. The study was based on three main components—filling a pre-intervention 10-minute self-report questionnaire, partaking in a 30-minute AR-VR training, and filling an identical post-intervention questionnaire, immediately after the training. The data was anonymized and collected via Qualtrics.

### Population and sample

Fifteen reserve paramedics who were requested to reinforce MDA after the initiation of the war participated in the training session, with a response rate of 80% (*n* = 12) to both measurements. Out of the participants, five had not participated in training or volunteering for six years. Two participants reported being inactive and without training for five years, one for four years, two for up to three years, one for two years, and one for one year.

### Tools

Assessment of six selected subjective variables was conducted using validated English measurement tools adapted for the purpose of this evaluation.

Self-efficacy was measured by using the original version of a scale developed by Schwarzer and Jerusalem (1995) [8]. Individual resilience was measured by the validated 10-item tool developed by Connor and Davidson (CD-RISC)) [9]. The above two scales were measured on a 5 scale likert scale (1- Not true at all, to 5- True nearly all the time). Additionally, perceived paramedic knowledge (4 items), perceived paramedic competency (5 items), and general intubation confidence (2 items) made use of an adapted version of a questionnaire by Waltrich et al., (2022) [6]. These three scales were also measured on a 5 scale likert scale (1- Strongly disagree, to 5- Strongly agree). Quality of training was adapted from a validated questionnaire from Kaim et al., (2023) [10] with 12 items.

Furthermore, demographic factors were collected, including gender, age, length of experience as a paramedic, date of last paramedic training, and the duration of unemployment from MDA.

### Procedure

Participants were informed upon their arrival to the training about the evaluation process and its purpose. Informed consent was requested from participants willing to partake in the evaluation process. The data was collected anonymously, following approval of the Ethics Committee of Tel Aviv University (number 0007375-2). Subsequently, participants were asked to complete the first round of data collection by completing the questionnaire. Upon the completion of the training, participants were asked to re-take the questionnaire, as well as to complete the Quality of Training questionnaire. For the sake of cross-referencing the responses, participants were asked to indicate a short, designated ID tag on their questionnaire in a manner that would allow matching of the data without compromising their anonymity.

### Statistical analysis

The statistical analysis of the results was performed using SPSS Version 29. The analysis included descriptive methods, due to the small sample size and nature of the pilot study. Prior to the analysis, indices were generated, and their reliability was assessed using Cronbach’s Alpha.

## Results

There was a notable enhancement in the average scores for all evaluated variables, with a significant increase in confidence in performing intubations and in personal resilience. In addition, the training’s quality was rated highly, achieving a score of 4.438 out of a possible 5 (See Table 1).

**Table 1:** Comparison of means, and their change per variable for the reserve paramedics following the trauma AR-VR training

Variables	Paramedic reserve training (*n* = 12)		
	Mean score before training (± SD)	Mean score after training (± SD)	Mean Change^a^
Self-Efficacy	3.917(± 0.611)	3.967 (± 0.619)	+ 0.05
Individual resilience	3.717(± 0.630)	4.008 (± 0.665)	+ 0.29
Perceived paramedic knowledge	3.778(± 0.808)	3.958 (0.871)	+ 0.18
Perceived paramedic competency	3.867(± 0.469)	4.017 (± 0.522)	+ 0.15
General intubation confidence	3.541(± 0.891)	3.833 (± 1.008)	+ 0.29
Quality of training		4.438 (± 0.419)	

^a^ Change in mean score was computed by subtracting the mean score prior to training from the one after the training

A high, significant correlation level was observed between self-efficacy and perceived paramedic knowledge (0.675, *p* < 0.05); perceived paramedic competency (0.645, *p* < 0.05); and perceived intubation confidence (0.616, *p* < 0.05). Furthermore, individual resilience also presented a significant correlation with all three examined variables: perceived paramedic knowledge (0.667, *p* < 0.05); perceived paramedic competency (0.607, *p* < 0.05); and perceived intubation confidence (0.680, *p* < 0.05)

## Discussion

In the aftermath of the critical events Israel experienced on October 7th, 2023, the deployment of AR-VR in the training of reserve paramedics has yielded improvement across all measured variables following the intervention among the small sample. Although reserve paramedics displayed a high baseline level of perceived paramedic knowledge, confidence, and competence, the AR-VR training presented an improvement in perceived proficiency, which may facilitate a smooth transition back into active prehospital duty after periods of inactivity. Notably, the most considerable gains were observed in the areas of individual resilience and general intubation confidence. While the findings are preliminary, the AR-VR training system may be valuable for training when quick deployment of personnel is necessary and training resources are diminished, as the case was following October 7th, when ‘all hands on deck’ were necessary.

The literature on simulation in medical training is extensive and underscores its effectiveness not only for individual skill acquisition but also for enhancing team performance during critical interventions [11]. Specifically, simulation has proven effective for teaching intubation, with a study showing that paramedic students trained on mannequin simulators achieve success rates comparable to those trained on human subjects in the operating room [12]. A systematic review published last year revealed that, while several studies have explored the use of Augmented Reality and Virtual Reality simulators individually for teaching and improving intubation skills, no published manuscript has yet employed a combined AR-VR or mixed reality approach [11]. In contrast, the trauma simulator used in the current study leverages advanced AR-VR technology, which not only simulates the real-world complexities that can arise during intubation but also provides instant feedback on patient management through the precise one-to-one correspondence between the physical and virtual environments. Furthermore, it immerses participants in the environmental setting where intubation may need to occur. In this case, the simulation replicates a war scenario, complete with video imagery of injured soldiers, the sounds of explosions, and a treatment setting amidst rubble.

The elevation in individual resilience scores observed is particularly vital, as paramedics often function in highly stressful situations where psychological endurance is crucial, alongside clinical expertise [13]. Furthermore, it should be noted that the training received positive feedback. This feedback suggests that participants found value in the AR-VR training’s engaging and educational qualities, although it needs to be considered with caution given that the enjoyment factor—commonly associated with ‘fun’ training experiences—may also play a role.

Given the positive outcomes observed, there is a strong case for the future integration of AR-VR technology into the foundational, de-novo training of paramedics. However, further investigation is needed to compare the efficacy of this modality of trainings with the current gold-standard methods. Moreover, the flexibility of the AR-VR system, which supports both proctored and self-directed learning, may make it a practical solution for training paramedics in diverse settings, from urban centers to remote areas [14].

The significant correlations between individual resilience and the trio of examined variables — perceived knowledge, competency, and confidence in intubation — following the training highlight the synergy among these traits. As paramedics grow in resilience, they also gain confidence in their abilities, which positively influences their perception of knowledge and overall competence. This is in line with previous research work conducted among healthcare professionals on the relationship between resilience, self-eficacy, and perceived competencies [7].

## Limitations

Though this preliminary study highlights the benefits of AR-VR training for paramedics, it also has important limitations. The small sample size in this pilot study may not represent the broader paramedic researve population, affecting the findings’ generalizability and statistical significance. Additionally, we did not evaluate whether the participants had any prior AR-VR experience or similar training where one of the modalities was utilized. While high Cronbach’s Alpha values indicate reliable measurements, the absence of a control group limits the ability to definitively attribute improvements to AR-VR training. These improvements could be due to the novelty of the technology or the “enjoyment” factor associated with such a training. The use of self-reported perceived measures could introduce bias. Nonetheless, this research lays the groundwork for further investigation into the efficacy of AR-VR training in paramedic education. Objective assessment of performance with the inclusion of a larger sample size is essential to validate the effectiveness of this technology. Additionally, longitudinal assessments are necessary to evaluate the long-term impact and retention of the training.

## Conclusions

In summary, the preliminary data indicate that AR-VR training can be a valuable method for enhancing paramedic readiness by improving self-efficacy, resilience, perceived confidence, knowledge, and competence. This approach is especially crucial during times of crisis when quick deployment is required and there is limited time for refresher courses. The events of October 7th and the subsequent period highlighted the urgent need to streamline and expedite the preparation and training for essential health professionals. This need extends beyond wartime scenarios to include a variety of adversities, such as natural disasters and other emergencies. These enhancements are vital for the provision of emergency medical services under stressful conditions, as demonstrated by the events during which this pilot study took place [6]. This investigation opens new avenues for exploring the potential of trauma AR-VR simulation technologies to advance medical education and training. While the current research focuses on reserve paramedics, the findings also suggest that further exploration is needed to assess the potential for incorporating AR-VR technology into the de-novo training of paramedics. The ability to simulate real-world complexities that may arise during intubation and provide instant feedback makes this technology a valuable addition to traditional training methods.

## Data Availability

The analyzed data will be made available to requesting researchers upon a reasonable request.

## Abbreviations

EMS: Emergency Medical Services
MDA: Magen David Adom
VR: Virtual Reality
AR-VR: Augmented Reality-Virtual Reality
